# 

**Published:** 1892-10-01

**Authors:** 


					"Kit. TWOPENCE.
Vol. XIII.?Oct. 1st, 1892.
2,tie General Post Office ai a Newspaper far
fusion in t^e United Kingdom and Abroad. J
A
4
No. 314. Vol. XIII ] Saturday,
Oc TorsF.il 1st, 1892.
.JrTwi
PENCE.

				

